# World TB Day — March 24, 2017

**DOI:** 10.15585/mmwr.mm6611a1

**Published:** 2017-03-24

World TB Day is recognized each year on March 24, which commemorates the date in 1882 when Dr. Robert Koch announced his discovery of *Mycobacterium tuberculosis*, the bacillus that causes tuberculosis (TB). World TB Day provides an opportunity to raise awareness about TB and the measures needed to tackle this devastating disease. In 2017, for the second year, CDC will join the global Stop TB Partnership in adopting the World TB Day theme “Unite to End TB.”

 In 2016, a total of 9,287 new TB cases occurred in the United States (incidence of 2.9 cases per 100,000 persons) (*1*), a decrease from the 2015 case count and incidence. This 2016 provisional case count represents the lowest number of TB cases recorded since reporting began in 1953. However, data suggest that current strategies will not be sufficient to reach the goal of U.S. TB elimination during this century (*2*).

CDC is committed to eliminating TB in the United States. This will require expanded initiatives, both in the United States and globally. These initiatives must maintain and strengthen existing strategies for diagnosing and treating persons with TB disease and also increase testing and treatment of persons with latent TB infection as outlined in CDC recommendations and the 2016 recommendation from the U.S. Preventive Services Task Force (USPSTF) (*3*,*4*). Additional information about World TB Day and CDC’s TB elimination activities is available on CDC’s TB website (https://www.cdc.gov/tb/worldtbday).
